# Aqua­bis­(4-methyl­benzene­sulfonato-κ*O*)(η^5^-penta­methyl­cyclo­penta­dien­yl)rhodium(III) monohydrate

**DOI:** 10.1107/S160053681300860X

**Published:** 2013-04-13

**Authors:** Christopher P. Roy, Pauline M. Boyer, Joseph S. Merola

**Affiliations:** a Department of Chemistry, Virginia Tech, Blacksburg, VA 24061, USA

## Abstract

The title half-sandwich rhodium(III) complex, [Rh(C_10_H_15_)(C_7_H_7_O_3_S)_2_(H_2_O)]·H_2_O, consists of a π-bonded penta­methyl­cyclo­penta­dienyl group, two σ-bonded tosyl­ate groups and an aqua ligand. The structure displays both inter- and intra­molecular O—H⋯O hydrogen bonding. The inter­molecular hydrogen bonding results in an extended helical chain along a 2_1_ screw axis parallel to *c*, due to hydrogen bonding from the coordinating water ligand to the lattice water mol­ecule and then to a sulfonate O atom of a different asymmetric unit.

## Related literature

Synthesis details are given in Boyer *et al.* (1996 ▶). For the structure of another penta­methyl­cyclo­penta­dienylmetal bis-tosyl­ate (CCDC: 821138), see: Zaitsev *et al.* (2008 ▶). For the characterization of other aquo compounds, see: Bergmeister *et al.* (1990 ▶; CCDC: 601561) and Luo *et al.* (1990 ▶; CCDC: 595047). A survey of the geometry and environment of water molecules in crystalline hydrates studied by neutron diffraction can be found in in Ferraris & Franchini-Angela (1972 ▶).
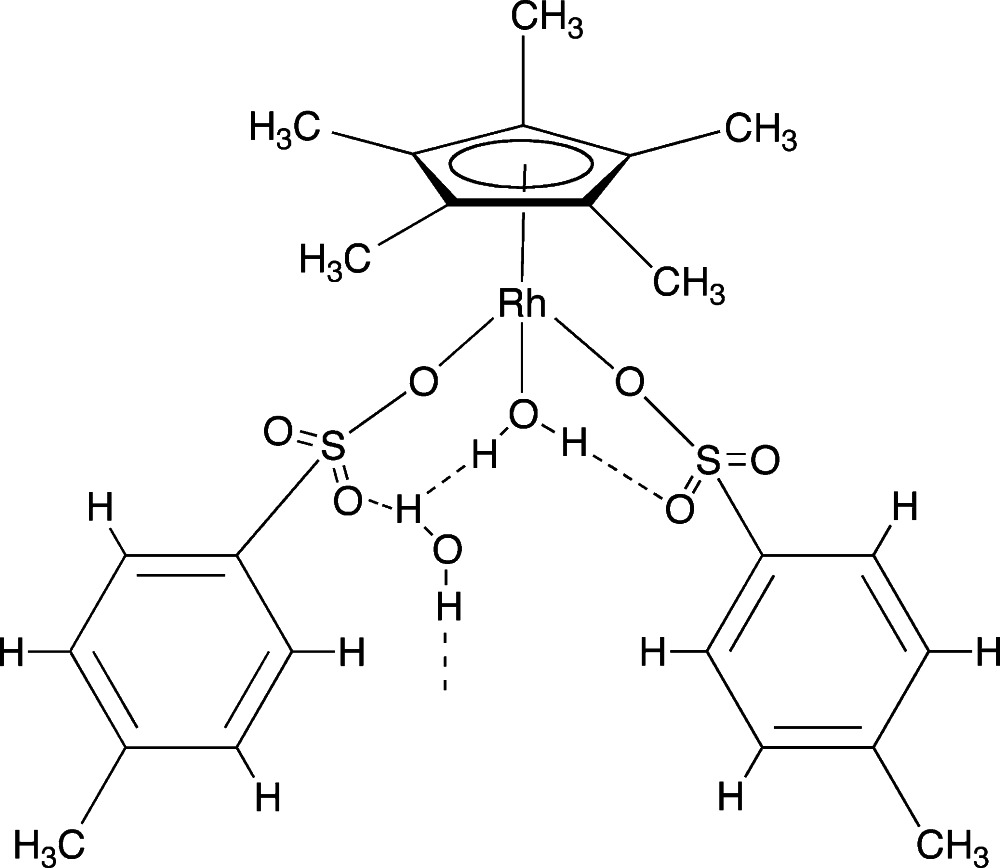


## Experimental

### 

#### Crystal data

[Rh(C_10_H_15_)(C_7_H_7_O_3_S)_2_(H_2_O)]·H_2_O
*M* *_r_* = 616.53
Orthorhombic, 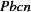

*a* = 23.550 (8) Å
*b* = 18.814 (7) Å
*c* = 12.114 (5) Å
*V* = 5367 (3) Å^3^
*Z* = 8
Mo *K*α radiation
μ = 0.84 mm^−1^
*T* = 295 K
0.4 × 0.4 × 0.4 mm

#### Data collection

Siemens P4 diffractometer
Absorption correction: ψ scan (North *et al.*, 1968 ▶) *T*_min_ = 0.00, *T*_max_ = 0.881
4738 measured reflections
4738 independent reflections
3297 reflections with *I* > 2σ(*I*)
3 standard reflections every 200 reflections intensity decay: 0(1)

#### Refinement

*R*[*F* ^2^ > 2σ(*F* ^2^)] = 0.038
*wR*(*F* ^2^) = 0.086
*S* = 1.03
4738 reflections
340 parameters
1 restraint
H atoms treated by a mixture of independent and constrained refinement
Δρ_max_ = 0.38 e Å^−3^
Δρ_min_ = −0.29 e Å^−3^

### 

Data collection: *XSCANS* (Siemens, 1994 ▶); cell refinement: *XSCANS*; data reduction: *XSCANS*; program(s) used to solve structure: *SHELXS97* (Sheldrick, 2008 ▶); program(s) used to refine structure: *SHELXL97* (Sheldrick, 2008 ▶); molecular graphics: *OLEX2* (Dolomanov *et al.*, 2009 ▶); software used to prepare material for publication: *OLEX2*.

## Supplementary Material

Crystal structure: contains datablock(s) I, global. DOI: 10.1107/S160053681300860X/pk2474sup1.cif

Structure factors: contains datablock(s) I. DOI: 10.1107/S160053681300860X/pk2474Isup2.hkl

Supplementary material file. DOI: 10.1107/S160053681300860X/pk2474Isup3.cdx

Additional supplementary materials:  crystallographic information; 3D view; checkCIF report

## Figures and Tables

**Table 1:** Hydrogen-bond geometry (Å, °)

*D*—H⋯*A*	*D*—H	H⋯*A*	*D*⋯*A*	*D*—H⋯*A*
O7—H7*A*⋯O8	0.85 (5)	1.99 (6)	2.608 (6)	128 (5)
O7—H7*B*⋯O3	1.11 (8)	1.64 (8)	2.647 (5)	147 (7)
O8—H8*D*⋯O2^i^	0.77 (8)	2.06 (8)	2.807 (6)	162 (8)
O8—H8*E*⋯O5	0.88 (6)	1.91 (6)	2.766 (7)	162 (6)

Symmetry code: (i) 
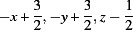
.

**Fig. 1.**
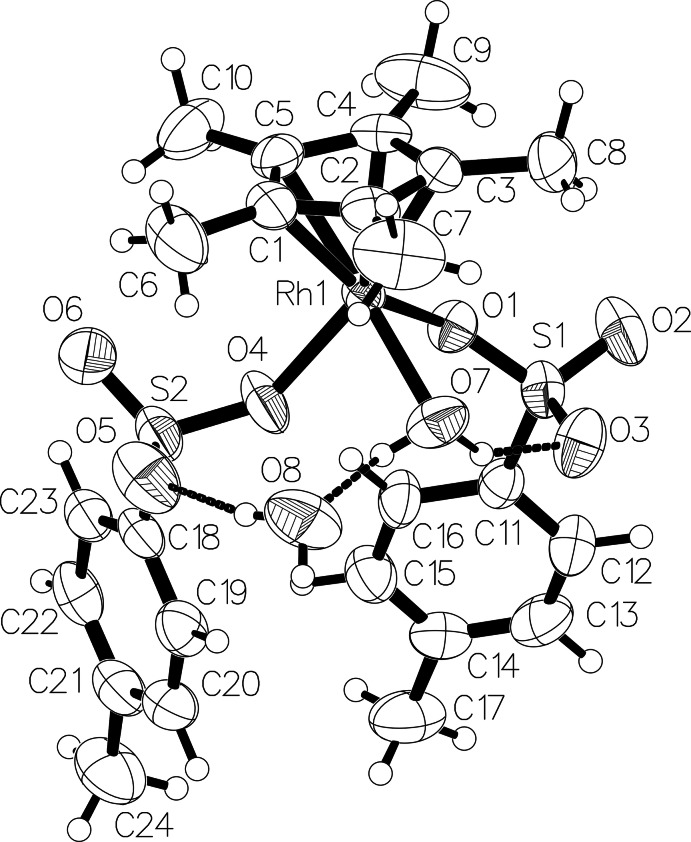
An ellipsoid plot (30% probability) view of the title compound.

**Fig. 2.**
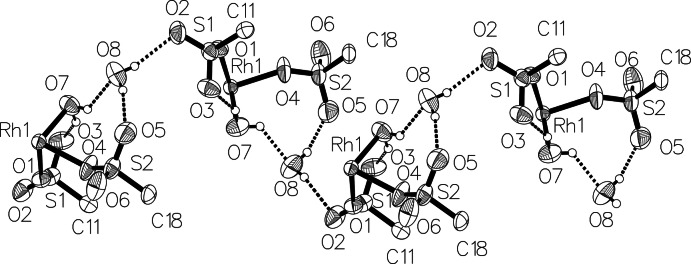
A view of the hydrogen-bonded helical chain that propagates parallel to the c-axis.

## supplementary crystallographic information

### Comment

The title compound adds to the body of organometallic compounds with water as a ligand. The empirically discovered requirement that water attached to metals must also be hydrogen-bonded either intermolecularly or intramolecularly still holds with this complex where the bonded water is H-bonded both intramolecularly to a sulfate oxygen and intermolecularly to a lattice water molecule. The hydrogen bonding creates a helical motif that runs parallel to the *c*-axis.

### Experimental

The title compound was prepared in a manner analogous to our previously reported carboxylate compounds using [(C_5_Me_5_)RhCl_2_]_2_ and silver tosylate. (Boyer *et al.*, 1996).

### Refinement

1. Fixed *U*_iso_ At 1.2 times of: H23 of C23, H13 of C13, H12 of C12, H19 of C19, H15 of C15, H16 of C16, H22 of C22, H20 of C20 At 1.5 times of: {H6A,H6B,H6C} of C6, {H17A,H17B,H17C} of C17, {H24A,H24B,H24C} of C24, {H8A, H8B,H8C} of C8, {H7C,H7D,H7E} of C7, {H9A,H9B,H9C} of C9, {H10A,H10B,H10C} of C10 2.a Aromatic/amide H refined with riding coordinates: C12(H12), C13(H13), C15(H15), C16(H16), C19(H19), C20(H20), C22(H22), C23(H23) 2.b Idealized Me refined as rotating group: C6(H6A,H6B,H6C), C7(H7C,H7D,H7E), C8(H8A,H8B,H8C), C9(H9A,H9B,H9C), C10(H10A, H10B,H10C), C17(H17A,H17B,H17C), C24(H24A,H24B,H24C)

### Crystal data

**Table:**

[Rh(C_10_H_15_)(C_7_H_7_O_3_S)_2_(H_2_O)]·H_2_O	*D*_x_ = 1.526 Mg m^−^^3^
*M**_r_* = 616.53	Mo *K*α radiation, λ = 0.71073 Å
Orthorhombic, *P**b**c**n*	Cell parameters from 50 reflections
*a* = 23.550 (8) Å	θ = 2–25°
*b* = 18.814 (7) Å	µ = 0.84 mm^−^^1^
*c* = 12.114 (5) Å	*T* = 295 K
*V* = 5367 (3) Å^3^	Prism, clear orange
*Z* = 8	0.4 × 0.4 × 0.4 mm
*F*(000) = 2544	

### Data collection

**Table:**

Siemens P4 diffractometer	3297 reflections with *I* > 2σ(*I*)
Radiation source: fine-focus sealed tube	*R*_int_ = 0.000
Graphite monochromator	θ_max_ = 25.0°, θ_min_ = 2.2°
profile data from θ/2θ scans	*h* = 0→28
Absorption correction: ψ scan (North *et al.*, 1968)	*k* = −22→0
*T*_min_ = 0.00, *T*_max_ = 0.881	*l* = 0→14
4738 measured reflections	3 standard reflections every 200 reflections
4738 independent reflections	intensity decay: 0(1)

### Refinement

**Table:**

Refinement on *F*^2^	Secondary atom site location: difference Fourier map
Least-squares matrix: full	Hydrogen site location: inferred from neighbouring sites
*R*[*F*^2^ > 2σ(*F*^2^)] = 0.038	H atoms treated by a mixture of independent and constrained refinement
*wR*(*F*^2^) = 0.086	*w* = 1/[σ^2^(*F*_o_^2^) + (0.0322*P*)^2^ + 3.4175*P*] where *P* = (*F*_o_^2^ + 2*F*_c_^2^)/3
*S* = 1.03	(Δ/σ)_max_ = 0.001
4738 reflections	Δρ_max_ = 0.38 e Å^−^^3^
340 parameters	Δρ_min_ = −0.29 e Å^−^^3^
1 restraint	Extinction correction: *SHELXL97* (Sheldrick, 2008), Fc^*^=kFc[1+0.001xFc^2^λ^3^/sin(2θ)]^-1/4^
Primary atom site location: structure-invariant direct methods	Extinction coefficient: 0.00052 (6)

### Special details

**Table:**

Geometry. All e.s.d.'s (except the e.s.d. in the dihedral angle between two l.s. planes) are estimated using the full covariance matrix. The cell e.s.d.'s are taken into account individually in the estimation of e.s.d.'s in distances, angles and torsion angles; correlations between e.s.d.'s in cell parameters are only used when they are defined by crystal symmetry. An approximate (isotropic) treatment of cell e.s.d.'s is used for estimating e.s.d.'s involving l.s. planes.
Refinement. Refinement of *F*^2^ against ALL reflections. The weighted *R*-factor *wR* and goodness of fit *S* are based on *F*^2^, conventional *R*-factors *R* are based on *F*, with *F* set to zero for negative *F*^2^. The threshold expression of *F*^2^ > σ(*F*^2^) is used only for calculating *R*-factors(gt) *etc*. and is not relevant to the choice of reflections for refinement. *R*-factors based on *F*^2^ are statistically about twice as large as those based on *F*, and *R*- factors based on ALL data will be even larger.

### Fractional atomic coordinates and isotropic or equivalent isotropic displacement parameters (Å^2^)

**Table:**

	*x*	*y*	*z*	*U*_iso_*/*U*_eq_	
Rh1	0.666388 (12)	0.645607 (15)	0.50870 (2)	0.03433 (11)	
S1	0.65268 (5)	0.82532 (6)	0.52292 (9)	0.0465 (3)	
S2	0.62414 (6)	0.60927 (6)	0.25074 (9)	0.0538 (3)	
O1	0.62955 (11)	0.75207 (14)	0.5237 (2)	0.0451 (7)	
O2	0.63499 (15)	0.86471 (18)	0.6196 (3)	0.0677 (10)	
O3	0.71402 (13)	0.82661 (17)	0.5072 (3)	0.0635 (9)	
O4	0.63324 (16)	0.65843 (16)	0.3441 (2)	0.0660 (10)	
O5	0.67723 (16)	0.57892 (19)	0.2134 (3)	0.0802 (11)	
O6	0.58036 (17)	0.55830 (19)	0.2723 (3)	0.0822 (11)	
O7	0.74332 (15)	0.6962 (2)	0.4484 (3)	0.0604 (9)	
H7A	0.736 (2)	0.681 (3)	0.384 (5)	0.09 (2)*	
H7B	0.730 (3)	0.753 (4)	0.442 (6)	0.17 (3)*	
O8	0.7836 (2)	0.6195 (3)	0.2870 (4)	0.0896 (15)	
H8D	0.802 (3)	0.632 (4)	0.238 (6)	0.13 (3)*	
H8E	0.754 (3)	0.602 (3)	0.252 (5)	0.09 (2)*	
C1	0.6733 (2)	0.5351 (2)	0.5370 (3)	0.0510 (12)	
C2	0.71567 (19)	0.5703 (2)	0.5983 (4)	0.0505 (11)	
C3	0.68878 (19)	0.6193 (2)	0.6720 (3)	0.0470 (11)	
C4	0.62904 (18)	0.6102 (2)	0.6596 (3)	0.0482 (11)	
C5	0.6187 (2)	0.5592 (2)	0.5754 (4)	0.0512 (12)	
C6	0.6836 (3)	0.4784 (3)	0.4526 (4)	0.097 (2)	
H6A	0.7128	0.4937	0.4027	0.146*	
H6B	0.6493	0.4697	0.4122	0.146*	
H6C	0.6954	0.4355	0.4889	0.146*	
C7	0.7786 (2)	0.5599 (4)	0.5867 (5)	0.097 (2)	
H7C	0.7981	0.6007	0.6154	0.145*	
H7D	0.7879	0.5539	0.5101	0.145*	
H7E	0.7899	0.5183	0.6271	0.145*	
C8	0.7184 (3)	0.6679 (3)	0.7509 (4)	0.0870 (19)	
H8A	0.7038	0.7153	0.7424	0.130*	
H8B	0.7584	0.6677	0.7358	0.130*	
H8C	0.7119	0.6520	0.8251	0.130*	
C9	0.5840 (3)	0.6498 (3)	0.7230 (5)	0.098 (2)	
H9A	0.5486	0.6468	0.6841	0.147*	
H9B	0.5950	0.6987	0.7303	0.147*	
H9C	0.5799	0.6290	0.7949	0.147*	
C10	0.5612 (2)	0.5339 (3)	0.5388 (5)	0.091 (2)	
H10A	0.5641	0.5131	0.4666	0.136*	
H10B	0.5354	0.5733	0.5366	0.136*	
H10C	0.5474	0.4989	0.5899	0.136*	
C11	0.62207 (17)	0.8673 (2)	0.4062 (3)	0.0427 (10)	
C12	0.6297 (2)	0.9397 (2)	0.3923 (4)	0.0576 (12)	
H12	0.6498	0.9656	0.4446	0.069*	
C13	0.6074 (2)	0.9732 (3)	0.3013 (4)	0.0639 (14)	
H13	0.6128	1.0219	0.2931	0.077*	
C14	0.5773 (2)	0.9366 (3)	0.2217 (4)	0.0554 (12)	
C15	0.5711 (2)	0.8643 (2)	0.2361 (4)	0.0571 (12)	
H15	0.5512	0.8383	0.1834	0.068*	
C16	0.59347 (19)	0.8292 (2)	0.3267 (4)	0.0510 (11)	
H16	0.5892	0.7802	0.3338	0.061*	
C17	0.5522 (2)	0.9735 (3)	0.1226 (4)	0.0834 (18)	
H17A	0.5754	0.9641	0.0589	0.125*	
H17B	0.5509	1.0238	0.1358	0.125*	
H17C	0.5145	0.9561	0.1098	0.125*	
C18	0.59902 (19)	0.6660 (2)	0.1437 (3)	0.0475 (11)	
C19	0.63637 (19)	0.7094 (3)	0.0871 (4)	0.0543 (12)	
H19	0.6749	0.7080	0.1035	0.065*	
C20	0.6164 (2)	0.7548 (3)	0.0061 (4)	0.0591 (12)	
H20	0.6419	0.7834	−0.0323	0.071*	
C21	0.5590 (2)	0.7586 (3)	−0.0189 (4)	0.0584 (12)	
C22	0.5227 (2)	0.7139 (3)	0.0370 (4)	0.0575 (13)	
H22	0.4842	0.7148	0.0200	0.069*	
C23	0.54202 (19)	0.6675 (2)	0.1184 (3)	0.0519 (12)	
H23	0.5167	0.6379	0.1553	0.062*	
C24	0.5368 (3)	0.8104 (3)	−0.1051 (4)	0.0871 (19)	
H24A	0.5559	0.8024	−0.1739	0.131*	
H24B	0.5437	0.8583	−0.0809	0.131*	
H24C	0.4967	0.8034	−0.1147	0.131*	

### Atomic displacement parameters (Å^2^)

**Table:**

	*U*^11^	*U*^22^	*U*^33^	*U*^12^	*U*^13^	*U*^23^
Rh1	0.03743 (17)	0.03487 (17)	0.03068 (16)	0.00144 (14)	−0.00110 (14)	0.00030 (14)
S1	0.0509 (6)	0.0401 (5)	0.0485 (6)	0.0032 (5)	−0.0098 (5)	−0.0090 (5)
S2	0.0766 (8)	0.0486 (7)	0.0362 (6)	0.0044 (6)	−0.0117 (6)	−0.0066 (5)
O1	0.0436 (16)	0.0396 (16)	0.0522 (17)	0.0031 (12)	−0.0028 (13)	0.0010 (13)
O2	0.092 (3)	0.060 (2)	0.0503 (18)	0.0172 (19)	−0.0182 (18)	−0.0173 (16)
O3	0.0477 (17)	0.0533 (18)	0.090 (2)	−0.0023 (15)	−0.0137 (18)	−0.0075 (19)
O4	0.113 (3)	0.049 (2)	0.0358 (16)	0.0138 (19)	−0.0241 (17)	−0.0100 (14)
O5	0.097 (3)	0.080 (3)	0.064 (2)	0.035 (2)	−0.008 (2)	−0.0091 (19)
O6	0.118 (3)	0.070 (2)	0.059 (2)	−0.027 (2)	−0.018 (2)	0.0126 (18)
O7	0.054 (2)	0.055 (2)	0.072 (2)	−0.0047 (17)	0.0159 (19)	0.0070 (19)
O8	0.087 (3)	0.119 (4)	0.063 (3)	0.008 (3)	0.025 (3)	−0.002 (3)
C1	0.084 (4)	0.029 (2)	0.040 (2)	0.010 (2)	−0.006 (2)	0.0060 (17)
C2	0.051 (3)	0.054 (3)	0.047 (2)	0.009 (2)	−0.007 (2)	0.015 (2)
C3	0.058 (3)	0.049 (3)	0.034 (2)	−0.010 (2)	−0.007 (2)	0.0047 (19)
C4	0.051 (3)	0.053 (3)	0.040 (2)	0.004 (2)	0.014 (2)	0.014 (2)
C5	0.055 (3)	0.052 (3)	0.047 (3)	−0.015 (2)	−0.011 (2)	0.017 (2)
C6	0.184 (7)	0.049 (3)	0.058 (3)	0.022 (4)	−0.008 (4)	−0.006 (3)
C7	0.059 (4)	0.129 (6)	0.102 (5)	0.037 (4)	0.003 (3)	0.030 (4)
C8	0.126 (5)	0.081 (4)	0.054 (3)	−0.043 (4)	−0.034 (3)	0.008 (3)
C9	0.103 (5)	0.116 (5)	0.075 (4)	0.048 (4)	0.046 (4)	0.023 (4)
C10	0.080 (4)	0.096 (4)	0.097 (4)	−0.043 (3)	−0.039 (3)	0.044 (4)
C11	0.043 (2)	0.036 (2)	0.049 (2)	−0.0010 (19)	0.0031 (19)	−0.0045 (19)
C12	0.062 (3)	0.047 (3)	0.064 (3)	−0.008 (2)	−0.006 (2)	−0.010 (2)
C13	0.074 (4)	0.043 (3)	0.074 (3)	−0.002 (3)	0.007 (3)	0.014 (3)
C14	0.051 (3)	0.065 (3)	0.050 (3)	0.002 (3)	0.008 (2)	0.011 (2)
C15	0.066 (3)	0.057 (3)	0.049 (3)	0.001 (2)	−0.011 (2)	−0.003 (2)
C16	0.063 (3)	0.034 (2)	0.055 (3)	−0.003 (2)	−0.010 (2)	−0.008 (2)
C17	0.086 (4)	0.091 (4)	0.073 (4)	−0.006 (3)	0.000 (3)	0.034 (3)
C18	0.059 (3)	0.054 (3)	0.029 (2)	0.002 (2)	−0.006 (2)	−0.0066 (19)
C19	0.045 (3)	0.070 (3)	0.047 (3)	−0.002 (2)	−0.006 (2)	−0.004 (2)
C20	0.065 (3)	0.069 (3)	0.043 (3)	−0.008 (2)	−0.002 (2)	0.004 (2)
C21	0.077 (3)	0.060 (3)	0.038 (2)	0.008 (3)	−0.008 (2)	−0.003 (2)
C22	0.052 (3)	0.073 (3)	0.047 (3)	0.009 (3)	−0.014 (2)	−0.015 (2)
C23	0.055 (3)	0.061 (3)	0.040 (2)	−0.007 (2)	−0.001 (2)	−0.008 (2)
C24	0.112 (5)	0.091 (4)	0.059 (3)	0.018 (4)	−0.021 (3)	0.010 (3)

### Geometric parameters (Å, º)

**Table:**

Rh1—O1	2.190 (3)	C8—H8B	0.9600
Rh1—O4	2.155 (3)	C8—H8C	0.9600
Rh1—O7	2.173 (3)	C9—H9A	0.9600
Rh1—C1	2.113 (4)	C9—H9B	0.9600
Rh1—C2	2.129 (4)	C9—H9C	0.9600
Rh1—C3	2.106 (4)	C10—H10A	0.9600
Rh1—C4	2.135 (4)	C10—H10B	0.9600
Rh1—C5	2.135 (4)	C10—H10C	0.9600
S1—O1	1.482 (3)	C11—C12	1.384 (6)
S1—O2	1.447 (3)	C11—C16	1.377 (6)
S1—O3	1.457 (3)	C12—H12	0.9300
S1—C11	1.773 (4)	C12—C13	1.373 (6)
S2—O4	1.476 (3)	C13—H13	0.9300
S2—O5	1.447 (4)	C13—C14	1.382 (6)
S2—O6	1.432 (4)	C14—C15	1.380 (6)
S2—C18	1.781 (4)	C14—C17	1.507 (6)
O7—H7A	0.85 (5)	C15—H15	0.9300
O7—H7B	1.11 (8)	C15—C16	1.386 (6)
O8—H8D	0.77 (8)	C16—H16	0.9300
O8—H8E	0.88 (6)	C17—H17A	0.9600
C1—C2	1.410 (6)	C17—H17B	0.9600
C1—C5	1.439 (6)	C17—H17C	0.9600
C1—C6	1.498 (6)	C18—C19	1.382 (6)
C2—C3	1.431 (6)	C18—C23	1.377 (6)
C2—C7	1.501 (6)	C19—H19	0.9300
C3—C4	1.425 (6)	C19—C20	1.384 (6)
C3—C8	1.495 (6)	C20—H20	0.9300
C4—C5	1.422 (6)	C20—C21	1.387 (7)
C4—C9	1.506 (6)	C21—C22	1.377 (7)
C5—C10	1.501 (6)	C21—C24	1.523 (6)
C6—H6A	0.9600	C22—H22	0.9300
C6—H6B	0.9600	C22—C23	1.394 (6)
C6—H6C	0.9600	C23—H23	0.9300
C7—H7C	0.9600	C24—H24A	0.9600
C7—H7D	0.9600	C24—H24B	0.9600
C7—H7E	0.9600	C24—H24C	0.9600
C8—H8A	0.9600		
			
O4—Rh1—O1	80.25 (11)	C1—C6—H6B	109.5
O4—Rh1—O7	86.66 (15)	C1—C6—H6C	109.5
O7—Rh1—O1	87.57 (13)	H6A—C6—H6B	109.5
C1—Rh1—O1	156.42 (15)	H6A—C6—H6C	109.5
C1—Rh1—O4	106.75 (14)	H6B—C6—H6C	109.5
C1—Rh1—O7	114.93 (17)	C2—C7—H7C	109.5
C1—Rh1—C2	38.81 (17)	C2—C7—H7D	109.5
C1—Rh1—C4	65.50 (17)	C2—C7—H7E	109.5
C1—Rh1—C5	39.60 (17)	H7C—C7—H7D	109.5
C2—Rh1—O1	141.46 (15)	H7C—C7—H7E	109.5
C2—Rh1—O4	138.06 (15)	H7D—C7—H7E	109.5
C2—Rh1—O7	90.48 (16)	C3—C8—H8A	109.5
C2—Rh1—C4	65.18 (17)	C3—C8—H8B	109.5
C2—Rh1—C5	65.60 (17)	C3—C8—H8C	109.5
C3—Rh1—O1	103.66 (15)	H8A—C8—H8B	109.5
C3—Rh1—O4	170.43 (15)	H8A—C8—H8C	109.5
C3—Rh1—O7	102.12 (16)	H8B—C8—H8C	109.5
C3—Rh1—C1	66.24 (16)	C4—C9—H9A	109.5
C3—Rh1—C2	39.49 (16)	C4—C9—H9B	109.5
C3—Rh1—C4	39.27 (16)	C4—C9—H9C	109.5
C3—Rh1—C5	66.26 (16)	H9A—C9—H9B	109.5
C4—Rh1—O1	92.95 (14)	H9A—C9—H9C	109.5
C4—Rh1—O4	132.68 (17)	H9B—C9—H9C	109.5
C4—Rh1—O7	140.18 (17)	C5—C10—H10A	109.5
C4—Rh1—C5	38.89 (17)	C5—C10—H10B	109.5
C5—Rh1—O1	117.19 (16)	C5—C10—H10C	109.5
C5—Rh1—O4	104.17 (15)	H10A—C10—H10B	109.5
C5—Rh1—O7	154.07 (17)	H10A—C10—H10C	109.5
O1—S1—C11	105.65 (18)	H10B—C10—H10C	109.5
O2—S1—O1	111.4 (2)	C12—C11—S1	118.9 (3)
O2—S1—O3	112.5 (2)	C16—C11—S1	121.6 (3)
O2—S1—C11	107.48 (19)	C16—C11—C12	119.4 (4)
O3—S1—O1	112.37 (17)	C11—C12—H12	120.0
O3—S1—C11	107.0 (2)	C13—C12—C11	120.0 (4)
O4—S2—C18	103.32 (18)	C13—C12—H12	120.0
O5—S2—O4	111.2 (2)	C12—C13—H13	119.1
O5—S2—C18	107.2 (2)	C12—C13—C14	121.8 (4)
O6—S2—O4	112.6 (2)	C14—C13—H13	119.1
O6—S2—O5	114.5 (2)	C13—C14—C17	121.9 (5)
O6—S2—C18	107.1 (2)	C15—C14—C13	117.2 (4)
S1—O1—Rh1	134.78 (17)	C15—C14—C17	120.9 (5)
S2—O4—Rh1	133.72 (19)	C14—C15—H15	119.0
Rh1—O7—H7A	89 (4)	C14—C15—C16	122.0 (4)
Rh1—O7—H7B	102 (4)	C16—C15—H15	119.0
H7A—O7—H7B	102 (5)	C11—C16—C15	119.5 (4)
H8D—O8—H8E	101 (6)	C11—C16—H16	120.3
C2—C1—Rh1	71.2 (2)	C15—C16—H16	120.3
C2—C1—C5	108.4 (4)	C14—C17—H17A	109.5
C2—C1—C6	125.4 (5)	C14—C17—H17B	109.5
C5—C1—Rh1	71.0 (2)	C14—C17—H17C	109.5
C5—C1—C6	126.1 (5)	H17A—C17—H17B	109.5
C6—C1—Rh1	127.0 (3)	H17A—C17—H17C	109.5
C1—C2—Rh1	70.0 (2)	H17B—C17—H17C	109.5
C1—C2—C3	108.5 (4)	C19—C18—S2	120.2 (3)
C1—C2—C7	126.0 (5)	C23—C18—S2	119.9 (4)
C3—C2—Rh1	69.4 (2)	C23—C18—C19	119.9 (4)
C3—C2—C7	125.4 (5)	C18—C19—H19	120.0
C7—C2—Rh1	125.3 (3)	C18—C19—C20	120.0 (4)
C2—C3—Rh1	71.1 (2)	C20—C19—H19	120.0
C2—C3—C8	125.9 (5)	C19—C20—H20	119.4
C4—C3—Rh1	71.5 (2)	C19—C20—C21	121.1 (5)
C4—C3—C2	107.1 (4)	C21—C20—H20	119.4
C4—C3—C8	127.0 (5)	C20—C21—C24	121.2 (5)
C8—C3—Rh1	125.0 (3)	C22—C21—C20	117.9 (4)
C3—C4—Rh1	69.3 (2)	C22—C21—C24	120.9 (5)
C3—C4—C9	125.5 (5)	C21—C22—H22	119.1
C5—C4—Rh1	70.6 (2)	C21—C22—C23	121.8 (4)
C5—C4—C3	109.0 (4)	C23—C22—H22	119.1
C5—C4—C9	125.4 (5)	C18—C23—C22	119.2 (4)
C9—C4—Rh1	124.9 (3)	C18—C23—H23	120.4
C1—C5—Rh1	69.4 (2)	C22—C23—H23	120.4
C1—C5—C10	127.6 (5)	C21—C24—H24A	109.5
C4—C5—Rh1	70.6 (2)	C21—C24—H24B	109.5
C4—C5—C1	106.9 (4)	C21—C24—H24C	109.5
C4—C5—C10	125.5 (5)	H24A—C24—H24B	109.5
C10—C5—Rh1	127.2 (3)	H24A—C24—H24C	109.5
C1—C6—H6A	109.5	H24B—C24—H24C	109.5

### Hydrogen-bond geometry (Å, º)

**Table:**

*D*—H···*A*	*D*—H	H···*A*	*D*···*A*	*D*—H···*A*
O7—H7*A*···O8	0.85 (5)	1.99 (6)	2.608 (6)	128 (5)
O7—H7*B*···O3	1.11 (8)	1.64 (8)	2.647 (5)	147 (7)
O8—H8*D*···O2^i^	0.77 (8)	2.06 (8)	2.807 (6)	162 (8)
O8—H8*E*···O5	0.88 (6)	1.91 (6)	2.766 (7)	162 (6)

Symmetry code: (i) −*x*+3/2, −*y*+3/2, *z*−1/2.
